# Effect of heat shock protein 70 polymorphism on thermotolerance in Tharparkar cattle

**DOI:** 10.14202/vetworld.2016.113-117

**Published:** 2016-02-03

**Authors:** Sandip Bhat, Pushpendra Kumar, Neeraj Kashyap, Bharti Deshmukh, Mahesh Shivanand Dige, Bharat Bhushan, Anuj Chauhan, Amit Kumar, Gyanendra Singh

**Affiliations:** 1 Division of Animal Genetics, Indian Veterinary Research Institute, Izatnagar, Uttar Pradesh, India; 2 Department of Animal Genetics and Breeding, Govind Ballabh Pant University of Agriculture and Technology, Pantnagar, Uttarakhand, India; 3 Division of Physiology and Climatology, Indian Veterinary Research Institute, Izatnagar, Uttar Pradesh, India

**Keywords:** cattle, heat tolerance, heat shock protein 70, polymorphism, Tharparkar

## Abstract

**Aim:**

Out of various members of heat shock protein (HSP) superfamily which act a molecular chaperon by binding to the denaturing protein thus stabilizing them and preserving their activity, HSP70 are of major importance in thermotolerance development. Thus, present investigation aimed at a screening of HSP70 gene for polymorphisms and possible differences in thermotolerance in Tharparkar breed of cattle.

**Materials and Methods:**

A 295 bp fragment of HSP70 gene was subjected to polymerase chain reaction-single-strand conformation polymorphism (SSCP) followed by sequencing of different SSCP patterns in 64 Tharparkar cattle. A comparative thermotolerance of identified genotypes was analyzed using heat tolerance coefficients (HTCs) of animals for different seasons.

**Results:**

Three SSCP patterns and consequently two alleles namely A and B were documented in one fragment of HSP70 gene. On sequencing, one single-nucleotide polymorphism with G > T substitution was found at a position that led to a change of amino acid aspartate to tyrosine in allele A. It was found that in maintaining near normal average rectal temperature, genotype AA was superior (p≤0.01). Genotype AA, thus, was found to be most thermotolerant genotype with the highest HTC (p≤0.01).

**Conclusion:**

The polymorphism at HSP70 is expected to be a potent determinant for heat tolerance in cattle, which may aid in selection for thermotolerance in cattle.

## Introduction

India is a vast country comprising different agroecological regions with diverse climate, natural vegetation resources, topography that possess almost all domesticated animal species and crops with different production systems. In a tropical country like India, temperature in the summer rises about 40-48°C which unquestionably is out of the comfort range of animals and causing stress. Stress represents the reaction of the body to stimuli that disturbs homeostasis, often with detrimental effects [1] which reflected in the failure to achieve genetic potential for production traits resulting a negative influence on the growth [2], lactation [3,4], reproductive performance [5,6], and immune functions [2,7] of the animals.

When a cell experiences environmental stress, it stops or at least slows down most of its original functions, such as transport processes, DNA, RNA and protein synthesis. However, there are proteins which preferentially expressed under these, restrictive conditions like slick hair gene [8], ATP1A1 [9,10], ATP1B2 [11] and heat shock proteins (HSPs). HSPs are highly conserved proteins activated by numerous physiological and physical stressors [12]. HSP acts as molecular chaperones by participating in the assembly of proteins without being part of the final protein structure [13] and confer thermotolerance and the ability of the cell to survive injury and oxidative stress [14] by reducing the accumulation of damaged or abnormal polypeptides within cells [15]. They perform a crucial role in intracellular transport by maintaining proteins in an inactive form and preventing protein degradation [16]. HSP70 is an essential molecular chaperone of primary importance to all mammalian cells. Extensive research indicates that HSP70 can act as a molecular chaperone and protects the cell against exposures to lethal heat shock, which is capable of denaturing proteins. HSP70 along with HSP27 and HSP90 proteins is predominantly anti-apoptotic [17] and the cytoprotective functions of HSP70 have been established in many organs such as intestine [18], brain [19] and kidney [20] as well as in embryo [21].

During their separate evolution from *Bos taurus*, zebu cattle (*Bos indicus*) has acquired genes that confer thermotolerance at the physiological and cellular levels. Once specific genes responsible for thermotolerance in zebu have been identified or mapped, breeding strategies such as marker assisted selection can be applied to further the exploitation of the zebu genotype for cattle production systems [22]. Adamowicz *et al*. [23] found a novel single-nucleotide polymorphism (SNP) in the 3’ untranslated region of HSP70 gene, whereas Li *et al*. [24] found five novel mutations in HSP70 gene and reported that some of the genotypes confer better thermotolerance. Lamb *et al*. [25] found 8 SNPs in HSP70 gene of different cattle breeds and deduced that 5 of them were related to Brahman ancestry.

Therefore, in the present study, polymorphisms of HSP70 were explored for thermotolerance effects in Tharparkar cattle.

## Materials and Methods

### Ethical approval

The experiment was conducted following the code of ethics for animal experimentation with prior approval by the Institute's Animal Ethics Committee constituted as per the article number 13 of the CPCSEA rules laid down by Government of India.

### Experimental animals

A total of 64 Tharparkar cattle (one of the best indigenous dairy breed) from Cattle and Buffalo Farm, LPM section, Indian Veterinary Research Institute, Izatnagar, Uttar Pradesh were randomly selected for the present investigation. The study was conducted in strict accordance with the code of ethics for animal experimentation and animals were handled gently and carefully.

Rectal temperature (RT) and respiration rate (RR) of each animal were taken twice in a day (10 am and 2 pm) for 3 successive days in each season, i.e., winter (January), spring (March), and summer (May).

### Genotyping of animals

About 10 ml of blood from jugular vein was collected from each animal under sterile conditions and stored at −20°C until use. Genomic DNA was isolated from blood as per standard phenol: chloroform extraction protocol [26].

Primers were designed based on sequence of HSP70 gene to amplify two separate fragments of HSP70 gene of length 220 bp and 295 bp at initial coding region (Table-1). Amplification of HSP70 gene was carried out using polymerase chain reaction (PCR) reaction mixture of 25 μl containing 2.5 μl ×10 Taq buffer, 2 μl of 25 mM MgCl_2_, 1 μl of 50 ng genomic DNA, 1 μl of each of 10 pM/μl forward and reverse primers, 0.8 μl of 10 mM deoxynucleotides mix, 1 μl of 1 U/μl *Taq* DNA polymerase and final dilution was made by adding 15.7 μl of nuclease free water. PCR was done with 5 min of initial denaturation at 95°C followed by 30 cycles of 60 s denaturation at 95°C, 45 s annealing at 65°C for Fragment I and at 60°C for Fragment II, 60 s extension at 72°C and 10 min of final extension at 72°C. Horizontal submarine (1.5% w/v) agarose gel electrophoresis was used to check the amplification.

**Table-1:** Primer sequences used to amplify HSP70 gene of Tharparkar cattle.

Fragment	Primers		Amplicon size	Position on HSPA1A mRNA
I	Forward	5′-AAACATGGCTATCGGCATCGACCT-3′	295	181-475
	Reverse	5′-AGGCTTGTCTCCGTCGTTGATGA-3′		
II	Forward	5′-CTAAGGTGCAGGTGAGCTACAAAG-3′	220	474-693
	Reverse	5′-TTGATGATCCTCAGCACGTTCAGC-3′		

HSP70=Heat shock protein 70

Polymorphism was screened using PCR-single strand conformation polymorphism (SSCP) technique. About 5 μl of PCR product was taken with 15 μl formamide dye (95% v/v formamide, 4% v/v 0.5 M ethylenediaminetetraacetic acid [EDTA], 0.025% w/v bromophenol blue and 0.025% w/v xylene cyanol) and mixed properly followed by denaturation at 95°C for 10 min then snap chilling on ice for 15 min. The mixture was loaded on 12% polyacrylamide gel (acrylamide/bis-acrylamide 29:1, w/w). Electrophoresis was carried out using ×1 tris-borate-EDTA (45 mM tris-borate/1 mM EDTA) at 130 V for 20 h at 4°C temperature. Band patterns were detected by silver staining [27]. The bands representing different SSCP patterns were purified and sequenced for all the genotypes by the Sanger's sequencing method from both forward and reverse directions for each sample.

### Temperature humidity index and heat tolerance coefficient

Temperature humidity index (THI) was recorded by taking daily mean temperature as the average of maximum and minimum temperatures of the day for 3 successive days of each season, i.e., winter, spring and summer using dry and wet bulb thermometer to record dry and wet bulb air temperatures 4 times a day (8 am, 11 am, 2 pm and 5 pm) for 3 consecutive days for each season under study then THI was derived for each day and expressed as:

THI = 0.72 (T_w_ + T_D_) + 40.6 [28],

Where, T_W_ is wet bulb air temperature (°C) and T_D_ is dry bulb air temperature (°C).

Heat tolerance parameters RT and RR were recorded at 10 am and 2 pm for 3 successive days of each season. The average RT (ART) and average RR (ARR) were calculated as the mean of twice daily recording data. To assess heat adaptability of animals, heat tolerance coefficient (HTC) was calculated [29], with the equation:

HTC = 100−10 (ART−38.3),

Where, ART is average rectal temperature of 10 am and 2 pm of 3 consecutive days for each season and 38.3 is normal RT of cattle (°C).

### Statistical analysis

The effect of season and genotype was analyzed on different RTs (10 am, 2 pm and average), HTC and ARR for the population under study. A comparison of least square means was performed by GLM procedure of SAS 9.3 using the following model as Y_ijkl_ = μ + S_i_ + G_j_ + e_ijk_, where Y_ijk_ is the observation of the trait; μ is overall mean; S_i_ is effect of i^th^ season; G_j_ is fixed effect of j^th^ genotype and e_ijk_ is random error.

## Results and Discussion

**Figure-1:**
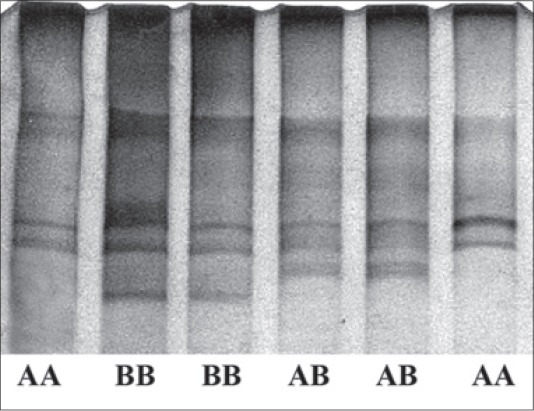
Polymerase chain reaction-single-strand conformation polymorphism patterns of Fragment I of heat shock protein 70 gene.

**Figure-2:**
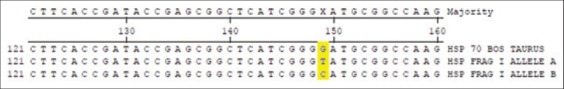
Sequence alignment of Fragment I of heat shock protein 70 gene.

**Figure-3:**
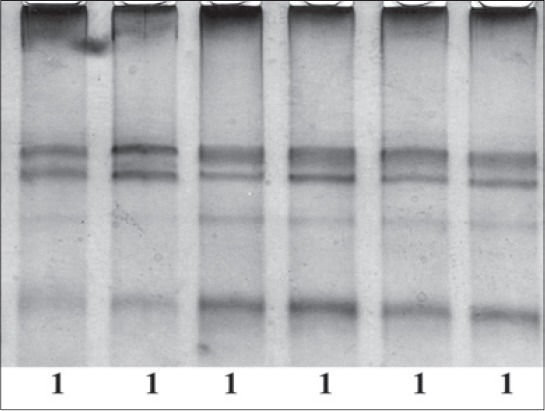
Polymerase chain reaction-single-strand conformation polymorphism patterns of Fragment II of heat shock protein 70 gene.

Tharparkar was found polymorphic for PCR-SSCP of amplified Fragment I (295 bp) of HSP70 gene and three SSCP-patterns were evident on PCR-SSCP (Figure-1). Nucleotide sequencing followed by alignment of three SSCP-patterns revealed a substitution of G > T and G > C at 149^th^ position of the amplicon with respect to *B. taurus* HSP70 gene (NM_174550.1), leading to two allele A with nucleotide T and allele B with nucleotide C (Figure-2) at 149^th^ position of Fragment I with 0.5078 and 0.4922 allele frequencies, respectively in Tharparkar population. The allele A and B were submitted to NCBI with accession no JX966362 and JX966363, respectively. Frequencies of three genotypes were 0.3281 for AA 0.3594 for AB and 0.3125 for BB. The SNP found was novel and has not yet been reported in any of the SNP databases for cattle. The SNP resulted in amino acid changes in A allele from aspartate to tyrosine whereas, in B allele aspartate changes to histidine with respect to *B. taurus* HSP70 protein. While the Fragment II (220 bp) of the HSP70 gene showed monomorphic SSCP pattern (Figure-3), which was further confirmed by random sample sequencing and submitted to NCBI with accession no JX966360.

During the study, the average THI values were the highest for summer (84.34) followed by spring (68.25) and lowest for winter (52.72) season. The THI in summer season exceeded 72, i.e., the reported threshold value of THI for dairy cattle, thus imposing heat stress. Least squares mean for genotype and season were significantly different (p≤0.01) for 10 am RT, 2 pm RT, ART and HTC (Table-2). In summer, the highest values for 10 am RT, 2 pm RT, ART and ARR while the lowest value for HTC in summer indicated heat stress. There were significant differences (p≤0.01) between genotypes with AA genotype holding lowest values for 10 am RT, 2 pm RT and ART in pooled seasons as well as in summer alone. Genotype AA held the highest value for HTC, followed by genotype AB (96.58±0.27) and BB (95.22±0.29). A significant genotype effect in ARR was recorded having highest value for genotype BB followed by AB while genotype AA accounted for the least ARR.

**Table-2:** Effects of genotype and season on heat tolerance parameters on Tharparkar cattle.

Effects	10 am RT	2 pm RT	ART	HTC	RR
Season					
Winter	38.17^a^±0.03	38.62^a^±0.03	38.39^a^±0.03	99.05^a^±0.29	14.88^a^±0.09
Spring	38.77^b^±0.03	38.85^b^±0.03	38.61^b^±0.03	96.84^b^±0.28	15.53^b^±0.11
Summer	38.64^c^±0.03	39.13^c^±0.03	38.89^c^±0.03	94.13^c^±0.29	17.3^c^±0.11
Genotype for all seasons					
AA	38.23^a^±0.03	38.72^a^±0.03	38.48^a^±0.03	98.22^a^±0.28	15.31^a^±0.08
AB	38.39^b^±0.03	38.88^b^±0.03	38.64^b^±0.03	96.58^b^±0.27	15.91^b^±0.07
BB	38.55^c^±0.03	38.99^c^±0.03	38.78^c^±0.03	95.22^c^±0.29	16.48^c^±0.07
Genotype for summer					
AA	38.52^a^±0.04	38.97^a^±0.05	38.74^a^±0.04	95.56^a^±0.41	16.74^a^±0.22
AB	38.63^b^±0.04	39.14^b^±0.04	38.88^b^±0.04	94.15^b^±0.39	17.24^b^±0.21
BB	38.78^c^±0.04	39.29^c^±0.05	39.03^c^±0.04	92.67^c^±0.42	17.93^c^±0.23

Different superscript in same column within season and genotype indicate significant differences (p≤0.01). HTC=Heat tolerance coefficient, RT=Rectal temperature, RR=Respiration rate, ART=Average rectal temperature, ARR=Average respiration rate

The effects of heat in the organisms are related to hyperthermia and consequent impairment of tissue and organ functions through reduction on blood flow [30]. The important physiological response of cattle to heat stress includes raised RRs and RT [31] that was evident in Tharparkar. In this study, the allelic variants of HSP70 gene were found to be associated with heat tolerability of the Tharparkar cattle. Li *et al*. [24], Basiricò *et al*. [32] and Xiong *et al*. [33] also found mutations at different locus in HSP70 gene in Holstein cattle that showed differences in thermotolerance. However, they were at different locus; they support the findings of the present study that the gene is important for thermotolerance in cattle. Further indigenous cattle like Tharparkar which have already been said to have thermotolerance, it has been confirmed by the present study that they may have more variations conferring thermotolerance that may provide a chance of improving these breeds for higher thermo-tolerability.

## Conclusion

Heat stress is one of the critical factors among various environmental stressors that impede profitable livestock rearing. The higher values of RT and RR in summer indicate heat stress in Tharparkar population. Present investigation establishes strong association between the HSP70 polymorphism and heat tolerability parameters, with the allele A having a positive effect on heat tolerance and genotype AA being superior in heat tolerance. Thus, this locus can be used as an indicator of thermotolerance in cattle, though further validation may be needed in other cattle breeds and in a larger population before its practical application.

## Authors’ Contributions

SB: Research was done by this author as the part of his master's degree thesis dissertation. PK: Designed the study and supervised the research as major advisor. NK, BD and MSD: Worked and collaborated in the lab work and compilation of the results as well as the manuscript. AC and AK: Provided valuable suggestions regarding the design of the experiment and analysis of the data collected during research. BB: advised in all aspects of the work and shared lab facilities. GS: As an expert of animal physiology and climatology, provided valuable suggestions and implementable ideas of recording physiological and climatological parameters. All authors read and approved the final manuscript.
